# Role of caregivers on medication adherence management in polymedicated patients with Alzheimer's disease or other types of dementia

**DOI:** 10.3389/fpubh.2022.987936

**Published:** 2022-10-24

**Authors:** María Cristina Muñoz-Contreras, Ignacio Segarra, Francisco Javier López-Román, Raúl Nieto Galera, Begoña Cerdá

**Affiliations:** ^1^Hospital Pharmacy, Hospital La Vega, Murcia, Spain; ^2^‘Pharmacokinetics, Patient Care and Translational Bioethics' Research Group, UCAM – Catholic University of Murcia, Murcia, Spain; ^3^Department of Pharmacy, Faculty of Pharmacy, UCAM – Catholic University of Murcia, Guadalupe, Spain; ^4^Health Sciences Department, UCAM – Catholic University of Murcia, Guadalupe, Spain; ^5^Biomedical Research Institute of Murcia (IMIB-Arrixaca), Murcia, Spain; ^6^AFAL Cartagena and Region, Cartagena, Spain; ^7^‘Nutrition, Oxidative Stress and Bioavailability' Research Group, UCAM – Catholic University of Murcia, Murcia, Spain

**Keywords:** adherence, Alzheimer, dementia, caregivers, gender, treatment satisfaction, polymedication

## Abstract

**Background:**

Alzheimer's disease (AD) and other dementia patients may have severe difficulties to ensure medication adherence due to their generally advanced age, polymedicated and multi-pathological situations as well as certain degree of cognitive impairment. Thus, the role of patient caregivers becomes crucial to warrantee treatment compliance.

**Purpose:**

To assess the factors associated to patients and caregivers on medication adherence of patients with AD and other types of dementia as well as the degree of caregiver satisfaction with respect to treatment.

**Methods:**

An observational, descriptive, cross-sectional study among the caregivers of 100 patients with AD and other types of dementia of the “Cartagena and Region Association of Relatives of Patients with Alzheimer's Disease and other Neurodegenerative Diseases” was conducted to assess patient and caregiver factors that influence medication adherence evaluated with the Morisky-Green-Levine test.

**Results:**

Overall, adherence to treatment was 71%, with similar proportions between male and female patients. Greater adherence was found in married or widowed patients (49.3%), first degree (85.9%) or female (81.7%) caregivers but lower in AD patients (75.9%). Multivariate analysis showed a statistically significant positive association between non-adherence and male sex of the caregiver (OR 3.512 [95%IC 1.124–10.973]), dementia (OR 3.065 [95%IC 1.019–9.219]), type of caregiver (non-first-degree relative) (OR 0.325 [95%IC 0.054–0.672]) and civil status of the patient (OR 2.011 [95%IC 1.155–3.501]) favorable for married or widowed patients. No or week association was found with gender, age, education level, number of drugs used or polymedicated status of the patient. Caregivers considered the use (90%) and administration (91%) of the treatment easy or very easy and rarely interfered with their daily life, especially for female caregivers (*p* = 0.016). Finally, 71% indicated that they were satisfied or very satisfied with the treatment received by the patient.

**Conclusions:**

Caregivers influence therapeutic management with predictors for improved adherence including female gender and first-degree kinship, together with patient's marital status. Thus, training caregivers about the disease and the importance of medication adherence in AD patients may ensure optimal treatment.

## Introduction

Dementia is a chronic and progressive syndrome in which there is a deterioration of cognitive functions, the ability to process thought, beyond what would be expected along normal aging (1). It is characterized by progressive short- and long-term memory loss and behavioral disturbances (2). Currently, dementia is one of the leading causes of death (3), as well as dependency and disability among people over 65 years of age, being a major global health problem that leads to increased morbidity and mortality (4). The total number of people affected with dementia is expected to reach 78 million by 2030 and 139 million by 2050 (5), a consequence of continued population aging that have led to increasing prevalence of neurodegenerative diseases (6).

Alzheimer's disease (AD) is presently the most common cause of dementia, accounting for 60 to 70% of cases (1), and there is currently no curative treatment. People with Alzheimer's disease have changes in blood-brain barrier permeability and endogenous neurotransmitter levels that increase the likelihood of drugs reaching the central nervous system (7–9). In addition, age-related physiological changes in the elderly lead to pharmacokinetic and pharmacodynamic alterations in the body, which increase sensitivity to drugs (8, 9). Thus, elderly people with Alzheimer's disease are extremely susceptible to medication-related problems (DRP) (8–10). This increased drug susceptibility of AD patients, coupled with the progression of cognitive impairment, frailty and the high prevalence of additional chronic diseases in this population results in the need for caregiver support (6, 11).

About 87.7% of people aged 62 to 85 years are prescribed a medication, of whom 35.8% have concomitant use of at least five medications (12). The complexity of pharmacological treatments prescribed to patients with dementia can cause problems of adherence to treatment, preventing the expected therapeutic benefits (13), hence the need for medication management and administration depend on the caregiver or immediate family member, playing a key role in the treatment of dementia (14).

Adherence to treatment, as defined by the World Health Organization (WHO), is the degree of a person's compliance in taking medication in accordance with the dosage schedule prescribed by a health professional (15). Hence, In dementia, polymedication and drug side effects are predictors of poor medication adherence (16–19). Lack of adherence can have serious health implications, including increased hospitalizations and worsening of disease (20). In general, it is estimated that 20–50% of patients do not take their medications as prescribed, and more specifically, non-compliance in the elderly with AD can range from 17 to 100% (21). Taking into account that, in general, this type of patients is multi-pathological and polymedicated patients and more prone to suffer drug-related problems, a multidisciplinary team is necessary to treat them and achieve the therapeutic objectives. In this sense, the pharmacist can monitor compliance with the patient's prescription, participate effectively in the identification of possible drug-related problems and provide information to patients and their caregivers.

Therefore, given the potential importance of non-adherence to treatment in these patients, the aims of this research were to assess the degree of adherence to treatment in patients with AD through the role of caregivers and to evaluate the degree of caregiver satisfaction with respect to treatment.

## Materials and methods

### Study design and population

An observational, descriptive, cross-sectional study was conducted (during 2018–2021) to assess the quality of care of these patients by means of treatment adherence and caregiver satisfaction. The study was developed in collaboration with the Cartagena and Region Association of Relatives of Patients with Alzheimer's Disease and other Neurodegenerative Diseases. This association provides support for this kind of patients and their families. Its objective is to improve the quality of life of patients through non-pharmacological treatments, as well as, to promote research and creation of social and health projects.

The study was reviewed and approved by the Institutional Ethics Committee of the Catholic University of Murcia (CE041808). An information sheet about the study was given to the caregiver together with the informed consent form. Those who agreed to the informed consent and met the following inclusion criteria were recruited and included in the study: male or female patient with a diagnosis of Alzheimer's disease or other type of dementia/neurodegenerative disease. The exclusion criteria were patient without a caregiver or family member who could act as a reliable assistant.

The pharmacological treatment was reviewed by means of the electronic prescription and updated medical reports. Finally, the pharmacist conducted a clinical interview with the person responsible for administering the medication.

### Variables and sources

Different types of sociodemographic and clinical variables of the patient and related to the caregivers were included in the study: age, sex, civil status, level of education, type of caregiver, gender of caregiver, type of dementia, number of prescribed medications and whether the patient was undergoing polymedication. Although there are various interpretations of polypharmacy, the most common one refers to the use of 5 or more different medications in the same person (22). The greater the number of medications a person takes and complexity of the treatment, the greater the incidence of poor adherence, adverse reactions or hospital admissions (23). This situation of polymedication is very common in older people as well as patients with chronic pathologies which in general lead to higher treatment burden and decreased adherence (24). Caregivers were considered either “informal,” usually a family member or close relative of the patient, or “formal,” a healthcare professional usually unrelated to the patient.

Adherence to treatment was evaluated with the Morisky-Green-Levine test (25) which has been used in other studies for this purpose (26–29). This test comprises of four questions with a dichotomous answer (yes/no) and to be considered “adherent,” the caregiver must answer “Yes” to question 2 and “No” to the other three questions. Their answers allowed to group the patient population in in two groups based on their adherence or lack of adherence (adherent and non-adherent groups).

The caregiver's satisfaction with the treatment was measured with an *ad hoc* questionnaire, similar to the ones used in study KAPPA (30) and the ENTERPRISE (26) studies, which assessed satisfaction with the use of rivastigmine patches *vs*. the oral route. The test consists of four questions which address the easiness of use of the treatment, the easiness to follow the administration regimen, the frequency with which the treatment interferes with the caregiver's daily life, and the overall satisfaction with the treatment. The measurement scale presents four response categories for each question: from easy to very difficult for questions 1 and 2, from always to never for question 3, and from satisfied to very dissatisfied for question 4 (**Figure 2**).

### Statistical analysis

A descriptive analysis of the data variables included in the study was performed. Qualitative variables were expressed as absolute frequency and their relative frequency in percentages. Continuous variables were evaluated to ensure that followed a normal distribution and were represented as mean ± standard deviation (SD).

The relationship of the variables under study with therapeutic adherence *vs*. non-adherence was analyzed using the Pearson Chi-square test for qualitative variables and the student's *t*-test to analyze quantitative variables with normal distribution (31).

Two logistic regression models of patient-related and caregiver-related variables, which were shown to be associated with the dependent variable adherence at a significant level *p* < 0.2 were included to assess the impact of each factor on medication adherence (32, 33). The independent effect of each explanatory variable, the odds ratio (OR) and their respective confidence intervals (95% CI) were used to assess the strength of their association with patient adherence to treatment in which case *p* < 0.05 was considered significant and normality was assessed previously using the Kolmogorov-Smirnov test. The data was processed using SPSS 23.0 for Windows^®^.

## Results

### Patient population features

A total of 100 patients were included in the study, 64% women and 36% men, with a mean age of 77.83 ± 10.14 and 81.56 ± 7.41 years for male and female patients, respectively (overall range: 42–95). Their civil status was mostly married (48%) or widow/widower (46%), 5% were single and 1% divorced. The educational level was diverse: the majority of participants had basic education (59%), 16% had secondary education (average), 9% had higher education (university) and 16% had no formal education.

Regarding their pathologies, most of the patients (61%) suffered from AD, 10% fronto-temporal dementia, 9% mixed dementia (AD and vascular dementia), 8% vascular dementia and 12% other types dementia. Comorbidities were present in most of the patients being hypertension (64%) and depressive syndrome (61%) the most frequent ones. In fact, 33% of the patients had between 1 and 3 comorbidities, 27% had between 4 and 5, 36% had more than 6 comorbidities and only 4% had no comorbidity. In addition, the mean number of drugs used during the chronic treatment phase was 7.7 ± 3.3 (range 2–17) with 82% of patients polymedicated (≥5 drugs) and 19% patients taking more than 10 different drugs in their pharmacological treatment concomitantly. Amongst the patients with AD, it was found that 13% had no specific treatment for dementia. The sociodemographic and clinical features of the study population are listed in Table 1 for the adherent and non-adherent patient groups.

**Table 1:** Prevalence of the adherence and non-adherence groups according to the results of the Morisky-Green-Levine test related to the sociodemographic factors of the patients^++^.

**Feature and descriptor**		**Patient adherence**		***p***
		**Yes (*n* = 71)**	**No (*n* = 29)**	
Patient gender	Male	39.4% (28)	27.6% (8)	0.263
	Female	60.6% (43)	72.4% (21)	
Age		79.63 ± 8.97	81.66 ± 7.73	0.190
Civil status of patient	Single	1.4% (1)	13.8% (4)	0.024
	Married	49.3% (35)	44.8% (13)	
	Divorced	0% (0)	3.4% (1)	
	Widow/widower	49.3% (35)	37.9% (11)	
Education level of patient^+++^	No studies	15.5% (11)	17.2% (5)	0.685
	Basic studies	62% (44)	51.7% (15)	
	Average studies	15.5% (11)	17.2% (5)	
	University studies	7% (5)	13.8% (4)	
Dementia	Alzheimer's disease	54.9% (39)	75.9% (22)	0.051
	Other dementia types	45.2% (32)	24.1% (7)	
N° drugs		7.77 ± 3.32	7.55 ± 3.32	0.562^+^
Polymedicated patient	Yes	83.1% (59)	79.3% (23)	0.655
	No	16.9% (12)	20.7% (6)	

+ Student t-test.

++ A license has been obtained from Dr. Morisky for use of the MMAS-4 scale.

+++ Basic studies would refer prior high school and average would include high school studies.

### Caregiver population characteristics

The large majority of patients (77%) had a female caregiver. The usual caregiver was a first degree relative (81%), either the son or the daughter (52%) or the spouse (29%) and in 6% of patients the caregiver was another family member. Last, a formal caregiver was in the 13% of patients. Unlike the patient population, the educational level of the caregivers was different: 30% had higher education studies, 23% had secondary education and 39% had basic education. Only 7% had no formal education studies. Table 2 shows these characteristics for the adherent and non-adherent patient groups.

**Table 2:** Prevalence of the adherence and non-adherence groups according to the results of the Morisky-Green-Levine test related to the features of the caregiver.

**Feature and descriptor**		**Patient adherence**		***p***
		**Yes (*n* = 71)**	**No (*n* = 29)**	
Type of caregiver	First degree relative	85.9% (61)	69% (20)	0.050
	Other	14.1% (10)	31% (9)	
Gender of caregiver	Male	18.3% (13)	34.5(10)	0.081
	Female	81.7% (58)	65.5% (19)	
Education level of caregiver^$^	No studies	5.6% (4)	10.3% (3)	0.449
	Basic studies	43.7% (31)	27.6% (8)	
	Average studies	21.1% (15)	27.6% (8)	
	University studies	28.2% (20)	34.5% (10)	

$ n = 70 for the adherent group as one non-respondent in the adherent group was removed.

### Medication adherence evaluation

**Figure 1:**
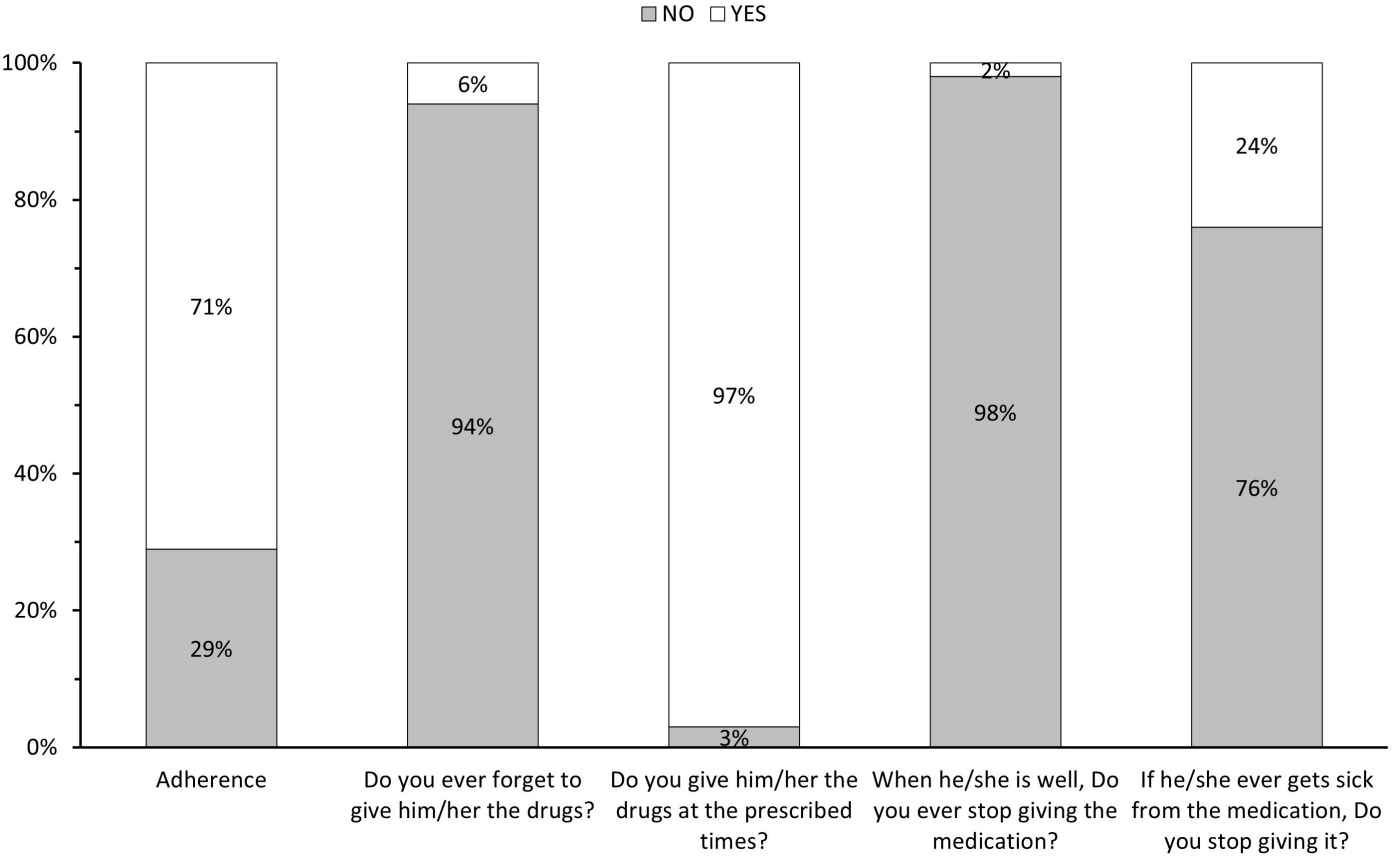
Results of the Morisky-Green-Levine test.

Medication adherence was measured with the Morisky-Green-Levine test (25) by the caregivers. The results showed that 29% of the patients did not adhere to their chronic treatment (Figure 1) with similar proportions between male and female patients (Table 1). Both patient (sociodemographic and clinical features) and caregiver (characteristics) related factors that affected non-adherence to treatment were also evaluated (Tables 1, 2, respectively).

The most important factors related to the patient which showed a stronger association were the patient's marital status (*p* = 0.025), with greater adherence (49.3%) in currently married or widowed patients (*p* = 0.024) and the type of dementia with lower adherence in patients with Alzheimer's disease (75.9%) *vs*. other types of dementia (*p* = 0.051). Regarding factors associated with the caregiver, it was found greater adherence (85.9%) when the caregiver was a first-degree relative (child or spouse) *vs*. other types of caregivers (*p* = 0.050) as well as the gender of the caregiver (*p* = 0.081) with greater patient therapeutic adherence (81.7%) with female caregivers. On the other hand, weak or no association seemed to exist with the sex (*p* = 0.359), age (*p* = 0.190) or educational level of the patient (*p* = 0.685), the number of chronic drugs used concomitantly (*p* = 0.727), the number of comorbidities (*p* = 0.553), being a polymedicated patient (*p* = 0.655) nor the educational level of the caregiver (*p* = 0.449).

Other items that were evaluated with the Morisky-Green-Levine test indicated that 97% of the caregivers gave the medication at the prescribed time, 98% of the caregivers did not stop giving the medication if the patient felt well, and 76% of caregivers stated that even if the patient felt ill, they never stopped giving it. On the other hand, 6% of the caregivers surveyed reported they would forget to give the medication to the patient (Figure 1). Lastly, in those patients who did not adhere to pharmacological treatment, 79.3% of caregivers would stop giving them the medication when the patient felt unwell.

### Caregiver satisfaction level

**Figure 2:**
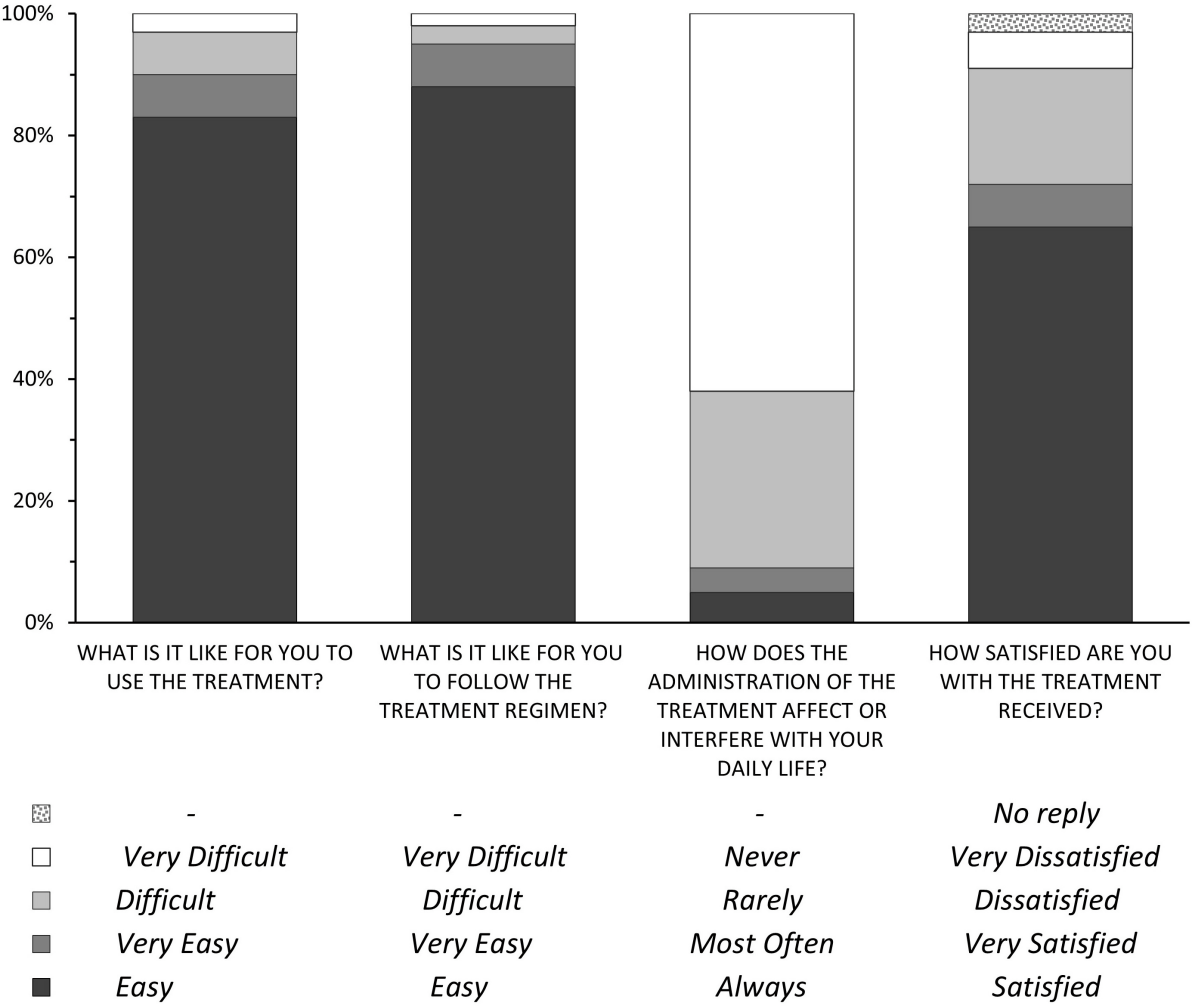
Results of the satisfaction survey.

The treatment satisfaction survey showed that 90% of the caregivers considered easy or very easy to use the medication, 95% found it easy or very easy to follow the treatment regimen, 91% indicated that the administration of the treatment never or rarely affected or interfered with their daily life, and 71% indicated that they were satisfied or very satisfied with the treatment received by the patient (Figure 2). Further statistical analysis showed possible association between sex of the caregiver (female) and satisfaction with the treatment received by the patient (*p* = 0.056) as well as the type of caregiver and the caregiver's use of the patient's pharmacological treatment, being easier to use among first-degree relatives than among others relatives (*p* = 0.007). In addition, statistically significant differences were observed between the sex of the caregiver and whether the administration of the treatment affected the caregiver's daily life, with lesser effects being observed in female caregivers (*p* = 0.016).

## Discussion

The present study evaluated the degree of treatment adherence of polymedicated patients with dementia and with several other pathologies through their caregivers as well as their degree of satisfaction. The sociodemographic and clinical data of the sample studied are similar to those of other studies with mean age of 76–77.2 years (14, 34–36), higher proportion of women around 60–65% (14, 34–36), educational level (37, 38), the caregiver was usually a family member (34) (child or spouse), and presents high presence of comorbidities (14) (hypertension). In general, the results showed non-adherence to treatment was 29% which was similar to values previously found which ranged from 10.7 to 38% (17).

There are several literature reviews on adherence to treatment in elderly patients with dementia (16, 17, 37–39) although mostly focused on the adherence to pharmacotherapy and the duration of the treatment rather than factors that could increase it (38) unlike the current study focused on adherence. Another study (16) identified factors contributing to non-adherence to medication including those related to the treatment, the patient, the health professionals, the disease itself, as well as socioeconomic factors. The results found in our study for the association between dementia or cognitive impairment and medication non-compliance are in agreement with their findings. In addition, it was observed that patients with Alzheimer's type dementia had lower adherence to treatment compared to patients with other types of dementia, e.g., vascular, frontotemporal, etc. This low adherence may be associated with the prescription of higher number of specific drugs (e.g., acetylcholinesterase inhibitors, NMDA receptor antagonists) and polymedication with the subsequent potential greater incidence of adverse effects (40). Both scenarios together with impaired cognitive function (41, 42) have been shown to be factors for non-adherence to treatment (20, 43, 44). On the other hand, another review (37) focused on the main barriers to adherence in patients with cognitive impairment, regardless the degree of impairment, and the interventions aimed to improve treatment adherence. Their results indicated a wide variety of barriers to treatment adherence, including inadequate communication with healthcare professionals, poor patient-caregiver relationships and difficulties scheduling logistics within care routines. However, it was observed that the scheduling of medication administration did not affect the caregiver's life and did not act as a barrier to adherence.

Overall, the results showed that 71% of the patients presented satisfactory treatment adherence, which is similar to other studies which measured adherence also through the patient's caregiver (27, 28). In a multicenter, cross-sectional, observational study (27) found a 63% treatment adherence, although they evaluated the degree of adherence to transdermal *vs*. oral formulation of rivastigmine, a different study goal from the current study but with similar values of adherence. Another similar study (28) observed a 70.2% degree of adherence to dementia drugs before and after a pharmaceutical intervention at a geriatric outpatient clinic of the university hospital.

The findings are in accordance with previous studies, and identified possible factors that may enhance treatment adherence, particularly those related to the caregiver. In fact, it has been suggested that high social support, and especially when it is provided by family members, improves adherence to treatment (45). Thus, when it was analyzed the role of the caregiver to enhance treatment adherence, a higher degree of adherence was observed when the caregiver was either female and/or a first-degree relative (Table 3), especially the spouse, probably associated with a higher dedication and affectivity due to a high personal affinity which seems to have a major role in monitoring the patient's medication providing high quality care (46). This is in contrast to other studies (36, 39, 47) where the patient-caregiver relationship or caregiver gender are not determining factors for better adherence to treatment or were considered to be included in the groups with lower adherence. Unlike the present study, which found that married patients have a higher degree of adherence to treatment. Other caregiver-related factors that have been shown to be strong determinants of medication adherence (17), whereby lower levels of cognitive functioning, self-efficacy, health literacy, and patient-provider relationship were significant characteristics of the lowest adherence group, in contrast to the present work, where the degree of kinship is associated with better adherence (Table 3).

**Table 3:** Logistic regression model for therapeutic adherence on caregiver level.

**Variable**	**Binary logistic regression**	
	**Adjusted OR (IC 95%)**	***p***
Gender of caregiver	3.692 (1.267–10.759)	0.017
Type of caregiver	0.231 (0.075–0.716)	0.011

The study demonstrates that the presence of a caregiver, especially when the caregiver is a first-degree relative, improves adherence to treatment (85.9%) in multi-pathological and polymedicated patients. A similar result was found in other study (48) which related the presence of a caregiver to better therapeutic adherence (83% vs. 65%; *p* = 0.005). It is worthwhile to note that these findings were achieved using different approaches. In the current study, adherence to treatment was evaluated using the Morisky-Green-Levine test, unlike their study which analyzed medication adherence in patients also with multiple pathologies using a validated questionnaire (49) for the identification of medication-related problems in users of a hospital emergency department (48). Thus, using different ways to measure it may be concluded that this factor (a first degree relative) may be crucial to reach around 80% treatment adherence. Also, it was found (50) that patients with a caregiver were 40% less likely to be non-adherent to their medications compared to patients without a caregiver, although the studies were not conducted with patients with dementia but cardiac patients, and therefore the role of the caregiver may be different. Besides, in their study, a patient was defined as adherent when the patient took more than 80% of all the doses of the prescribed medication in the last week (48), a different criterion.

Conversely, the multivariate analysis identified the male gender of the caregiver and having a caregiver who is not a first-degree relative (Table 3), the Alzheimer's disease type of dementia, the single and/or divorce marital status (Table 4) were predictor variables of potential therapeutic non-adherence. This gender gap has been reported also in different studies: a statistically significant association between non-adherence and male caregivers was found among caregivers of children undergoing anti-tuberculosis treatment (29) using also a multivariate analysis. Furthermore, these results, as other studies (47), may support the hypothesis that certain characteristics of the caregiver, such as male gender or degree of kinship with the patient may contribute to reduced and poor patient compliance.

**Table 4:** Logistic regression model for therapeutic adherence on patient level.

**Variable**	**Binary logistic regression**	
	**Adjusted OR (IC 95%)**	***p***
Dementia^*^	2.879 (1.039–7.979)	0.042
Civil status	1.762 (1.070–2.904)	0.026
Age of patient	0.946 (0.885–1.011)	0.100

* Dementia: (AD vs. other dementia types).

Finally, the results show greater satisfaction with the patient treatment by female caregivers. In addition, first-degree family caregivers find the usage of the patient's prescribed treatments easy. Approximately three quarters of the caregivers are satisfied with the treatment received by the patients and 95% of them consider it easy or very easy to follow the prescribed treatment regimen. This factor seems to be intrinsic to the caregiver as it is consistent with another study focused on adherence and satisfaction in patients with hypertension (51). Furthermore, dementia is a disease that affects the well-being of caregivers (52), and the inclusion of the caregivers' perspective is necessary to gain a better understanding of the experience of those living with dementia as included in the study.

### Perspective of the study

The increase in life expectancy will result in a larger aging population, with increasing cases of dementia, most likely with multiple pathologies and therefore polymedicated. Since these factors may be contributors to lack of adherence (20, 42, 44), treatment adherence may become a serious public health problem and a challenge for health systems to ensure optimal treatment. Furthermore, the increasing physical and emotional caregiver's burden (53), on whom it depends, in most cases, to provide the correct pharmacological treatment to the patient on a daily basis, open new challenges to ensure treatment adherence.

Therefore, it is necessary to highlight those factors that predict non-adherence in this type of patients and thus increase attention to them and their caregivers (13, 54), so that they can receive more help on the quality and safety of pharmacological treatment. This in turn, may lead to a greater patient-centered approach which would ensure better treatment adherence as seen with the type and gender of caregiver (e.g., a first degree relative *vs*. other types of relation and female gender) (55–57) as well as to develop future working hypothesis to better understand and enhance their care, both patients and caregivers. Of particular interest would be addressing the needs and features of caregivers, whether formal or informal, to enhance patient care through longitudinal, interventional studies along the course of the pathology which could prolong several years as life expectancy increases.

### Limitations

The study has several limitations that also require their analysis. It is a cross-sectional study with a large sample but limited to fully understand the impact on clinical events that may take place. The use of an indirect method for measuring adherence has been used extensively in the past in contrast to a direct method (pharmacological follow-up) or another indirect method (tablet count) may not only overestimate adherence but may not ensure that the patient has been taking the medication. However, the impact of these limitations may be small since the assessment of the adherence is not through the patient themselves but the caregiver whose role is to ensure taking the medication. On the other hand, other the possible variables that could affect patient adherence, such as the caregiver's level of knowledge of the pathologies or the medication or the route of administration which could influence patient care were not evaluated. A much larger sample size could bring forward the significance of other variables that may affect adherence.

In summary, factors such as female gender of the caregivers, the patient's marital status and the degree of kinship of caregivers with the patient, may improve adherence to treatment in patients with dementia. Thus, there may be other factors which could be addressed in future studies including communication skills, specific training and competence of caregivers, which have not been address in the current study. Attention should be focused on predictors of adherence/non-adherence and train and educate caregivers about the importance of their role end dedication to ensure adherence in patients with dementia, especially as most of the patients may be multi-pathological, polymedicated and with some degree of cognitive impairment. In addition, the relationship between the degree or cognitive impairment of the patients and adherence was not addressed in this study and remains an essential question for further exploration.

## Data availability statement

The original contributions presented in the study are included in the article/supplementary material, further inquiries can be directed to the corresponding author/s.

## Ethics statement

The studies involving human participants were reviewed and approved by Institutional Ethics Committee of the Catholic University of Murcia (CE041808). The patients/participants provided their written informed consent to participate in this study.

## Author contributions

MM-C, BC, and IS conceived and designed the study. MM-C and RG carried out participants recruitment and data collection. MM-C and FJL-R performed the statistical analysis. MM-C, BC, and IS carried out the data analysis and interpretation and wrote and edited the manuscript. All authors read and agreed to the final version of the manuscript. All authors contributed to the article and approved the submitted version.

## Conflict of interest

The authors declare that the research was conducted in the absence of any commercial or financial relationships that could be construed as a potential conflict of interest.

## Publisher's note

All claims expressed in this article are solely those of the authors and do not necessarily represent those of their affiliated organizations, or those of the publisher, the editors and the reviewers. Any product that may be evaluated in this article, or claim that may be made by its manufacturer, is not guaranteed or endorsed by the publisher.
